# Findings from a Feasibility Study of an Adapted Cognitive Behavioral Therapy Group Intervention to Reduce Depression among LGBTQ (Lesbian, Gay, Bisexual, Transgender, or Queer) Young People

**DOI:** 10.3390/jcm8070949

**Published:** 2019-06-29

**Authors:** William J. Hall, Benjamin Ruiz Rosado, Mimi V. Chapman

**Affiliations:** School of Social Work, University of North Carolina, Chapel Hill, NC 27599-3550, USA

**Keywords:** lesbian, gay, bisexual, transgender, queer, youth, depression, intervention, cognitive behavioral therapy, group psychotherapy

## Abstract

Depression disproportionately affects LGBTQ (lesbian, gay, bisexual, transgender, or queer) adolescents and young adults. Cognitive behavioral therapy (CBT) is an evidence-based treatment approach; however, there has been limited work to adapt and evaluate CBT with LGBTQ young people. This study examined the feasibility of an intervention called Being Out With Strength (BOWS), which is an 8-session, small-group, CBT-based intervention to reduce depression among LGBTQ young people. We used a descriptive cross-sectional mixed-methods feasibility study design to evaluate the feasibility of BOWS. Survey data were collected from 79 LGBTQ young adults, and interview data were collected from nine mental health professionals. Almost half of the young adults had clinically significant depressive symptoms. All providers indicated depression as a problem facing this population and a need for BOWS. Two-thirds of young people were interested in participating in BOWS. Providers believed that BOWS would be acceptable for LGBTQ-identified individuals, those in late adolescence or early/young adulthood, and those with mild or moderate depression. Youth and providers also made implementation recommendations concerning settings to implement BOWS, times of day for BOWS sessions, number of sessions, group size, and facilitator composition. There is a demand for BOWS, and it is likely acceptable for the target population. Study findings can be used in the future to successfully implement BOWS and evaluate preliminary efficacy.

## 1. Introduction

Depression is a debilitating and burdensome mental health disorder that disproportionately affects young people who identify as lesbian, gay, bisexual, transgender, or queer (LGBTQ) [1,2,3,4]. Recent findings from a nationally representative U.S. survey demonstrate that 60% of LGB young people felt sad or hopeless almost every day for at least 2 weeks in the past year, compared to 26% of heterosexual young people [3]. Regarding gender identity, 53% of transgender young people felt sad or hopeless almost every day for at least 2 weeks in the past year, compared to 30% of cisgender young people [4]. Depression can contribute to a number of impairments and life disruptions, including missing school or work [5,6,7], tension in interpersonal relationships [8,9,10,11], substance use or abuse to self-medicate [12,13,14], and chronic health conditions (e.g., cardiovascular disease, diabetes, and HIV/AIDS) [15,16]. In addition, among all mental health disorders, depression is most often associated with suicide [17]. Research also shows that LGBTQ young people are at increased risk for suicide. Compared to their heterosexual peers, LGB young people are about three times more likely to have thought about suicide (15% vs. 43%, respectively), three times more likely to have made a plan for suicide (12% vs. 38%), and five times more likely to have attempted suicide (7% vs. 35%) [4]. Similarly, compared to their cisgender peers, transgender young people were about three times more likely to have thought about suicide (16% vs. 44%, respectively), three times more likely to have made a plan for suicide (13% vs. 39%), and five times more likely to have attempted suicide (6% vs. 29%) [3].

High rates of depressive symptoms among LGBTQ people are attributed to the social stigma and stress directed at this population [18,19,20,21]. A recent national study found that 85% of LGBTQ young people reported experiencing verbal harassment at school in the past year and 16% were physically assaulted (e.g., punched, kicked, or injured with a weapon) [22]. In addition to aggression, many LGBTQ young people also experience rejection from loved ones after coming out. A national study found that 39% of LGBTQ people reported that a close friend or family member rejected them because of their sexual orientation or gender identity [23]. Unfortunately, many LGBTQ young people experience hostility and rejection from the most important people in their lives—their parents, friends, and peers. In the face of hostile environments, many young people decide to conceal or minimize their LGBTQ identity to prevent experiences of violence and rejection [20]. Managing visibility and openness about one’s sexual or gender identity entails an ongoing process of assessing individuals and environments for safety, considering the positive and negative consequences of being out or not, and determining how out to be and with whom. This decision process requires considerable mental energy and vigilance, which may be burdensome for LGBTQ young people [20]. Finally, some LGBTQ young people have internalized negative sociocultural views about themselves, such as viewing their identities as abnormal, immoral, or pathological. This negative internalization is commonly referred to as internalized homophobia or internalized transphobia and broadly as internalized oppression [24,25,26,27,28]. Given that LGBTQ young people face a unique set of risk mechanisms that contribute to the high rates of depression in this population, mental health interventions must be adapted to specifically address these stressors. 

Cognitive behavioral therapy (CBT) is a leading evidence-based treatment approach for depression [29,30]. However, extensive effort has not been taken to comprehensively adapt and rigorously evaluate CBT with LGBTQ people or LGBTQ young people in particular. To the authors’ knowledge, only one randomized controlled trial has been conducted with sexual minority young people to test a CBT-based intervention targeting mental health outcomes—ESTEEM (Effective Skills to Empower Effective Men) [31,32,33]. Although the outcome evaluation findings are encouraging in terms of reduced depressive symptoms, alcohol use problems, and sexual risk behaviors, ESTEEM (*N* = 63) was conducted in New York City and designed specifically for gay and bisexual men aged 18–35 years as the target population [33]. Therefore, an intervention gap remains for LGB women as well as queer, transgender, and genderqueer young people—individuals who often experience even higher rates of depressive symptoms, compared to cisgender gay and bisexual men [1,34,35]. A cohort pilot study of a CBT-based intervention for LGBTQ adolescents aged 15–18 years in Toronto, Canada called AFFIRM (*N* = 30) also showed significant reductions in depressive symptom severity from baseline to posttest and follow-up, as well as high acceptability of the intervention [36,37]. Despite the promising results from these two studies, additional research is needed concerning the feasibility, acceptability, and efficacy of CBT-based interventions targeting depression among LGBTQ young people. In addition, it is not known if such interventions are feasible outside of large urban areas in the Northeast, which typically have more positive social climates and more resources for LGBTQ people [38,39,40].

## 2. Study Objective

The overall objective of this study was to examine the feasibility of an intervention that was initially developed but not yet evaluated called Being Out With Strength (BOWS), which is an 8-session, manualized, small-group, CBT-based intervention aimed at reducing depressive symptoms among LGBTQ young people. BOWS is similar yet different from the ESTEEM and AFFIRM interventions noted above (see Table 1). BOWS primarily focuses on internalized oppression as the intervention target, which research shows significantly contributes to depression in LGBTQ people [25,27,41,42]. In addition, this study aims to fill a gap in the literature by examining the feasibility of an LGBTQ-focused mental health intervention in the southeastern United States, a region that is considerably different from large urban areas in the Northeast.

Findings from feasibility studies can be used to determine if an intervention should be subjected to efficacy testing and to inform the design and implementation of a future randomized controlled trial of an intervention [43]. The research questions for this feasibility study focusing on the BOWS intervention included: (1) Is there a demand for this intervention? (2) Is the intervention acceptable for the target population? (3) What are potential implementation issues that should be addressed if the intervention is to be successfully delivered? 

## 3. Methods

### 3.1. Study Design

A descriptive cross-sectional mixed-methods feasibility study design was used because we were in the initial phase of intervention development with BOWS. According to Bowen and colleagues [43], feasibility studies are needed when there are very few prior studies using a specific intervention approach and when extant research suggests that the target population needs unique consideration. The study design was based on feasibility concepts and methods outlined by Bowen and colleagues for behavioral health interventions [43]. We aimed to examine three key feasibility constructs: *demand* (i.e., the extent that there is a need for an intervention), *acceptability* (i.e., the extent that an intervention is attractive to program deliverers and intended recipients), and *implementation* (i.e., the extent that an intervention can be successfully delivered as proposed) [43]. To assess demand, acceptability, and implementation, the study involved purposive sampling of intended recipients (i.e., LGBTQ young people) and those who would deliver the intervention (i.e., mental health service providers), cross-sectional survey and interview data collection, and descriptive quantitative and qualitative analyses that would inform potential future pilot testing of the intervention. Approval for this study was obtained from the Institutional Review Board at the authors’ university.

### 3.2. Study Setting and Participants

The study was conducted in the Triangle region of North Carolina, which is located in the center of the state and includes Wake, Durham, and Orange counties. This area was selected for several reasons: its proximity to the authors’ university; it includes urban, suburban, and rural areas; diversity in terms of race/ethnicity; and social cultures that range from affirming to rejecting of LGBTQ people. Data were collected from two groups of participants using purposive sampling: LGBTQ young people and mental health service providers.

#### 3.2.1. Young Adult Participants

Young people were recruited via a posting on a Facebook group of LGBTQ college students and alumni from a local university, postings in the personal ads on Craigslist for men seeking men and women seeking women in the Triangle area, and flyers at 3 local coffee shops. Postings stated the researchers were seeking feedback from LGBTQ young people about a program to promote mental health. The postings directed potential participants to the website of the online survey, and the postings were displayed for 30 days while the survey was live. To be included in the study, youth had to identify as LGBTQ and be aged 18–29 years. Those who identified as heterosexual, questioning, or asexual were excluded. Informed consent was attained online before youth started taking the survey. The young adult sample included 79 participants. The ages of respondents ranged from 18 to 28 years with an average age of 22.1 years (SD = 2.4). For race/ethnicity, 72% of the respondents were White, 10% were Asian, 8% were Black/African American, 6% were multiracial, and 4% were Latinx. For sex assigned at birth, 72% reported female and 28% reported male. For gender identity, 45% identified as cisgender female; 30% identified as transgender, genderqueer, and/or gender non-binary; and 24% identified as cisgender male. For sexual orientation, 33% of the respondents identified as gay/lesbian; 19% identified as bisexual; 15% identified as queer; 15% identified as a combination of bisexual, pansexual, and/or queer; 9% identified as pansexual; and 9% identified as gay/lesbian and queer. For education level, 52% had some college education, 30% had a 4-year college education, 14% has some graduate level education, and 4% had a high school level of education.

#### 3.2.2. Service Provider Participants

Mental health service providers were recruited from a listing on an LGBTQ community center website of affirming counselors and psychotherapists in the Triangle region. Providers’ websites and bios were examined so that we could target those with experience working with LGBTQ youth, using CBT in their practice, and doing group counseling or psychotherapy. Providers who had all three of these areas of experience were emailed and invited to participate in the study on a rolling basis. Once interviews were scheduled, each provider was emailed the BOWS intervention manual to review prior to the interview. All interviews were one-on-one in person, and informed consent was attained before each interview began, led by the first author. The provider sample included 9 participants. The ages of providers ranged from 27 to 46 years with an average age of 35.4 years (SD = 6.6). For race/ethnicity, 78% of respondents were White, 11% were African American, and 11% were Asian. For sex assigned at birth, 67% reported female and 33% reported male. For gender identity, 56% identified as cisgender and 44% identified as transgender, genderqueer, genderfluid, and/or gender non-conforming. For sexual orientation, 56% identified as heterosexual and 44% identified as lesbian, gay, queer, and/or pansexual. For education level, 78% had a master’s degree and 22% had a doctoral degree. In terms of provider type, 5 were counselors, 2 were clinical social workers, 1 was an art therapist, and 1 was a clinical psychologist. The years of practice experience ranged from 4 to 20 with an average of 12.1 (SD = 5.5).

### 3.3. Intervention Description

The first author began developing BOWS in 2013 as part of a doctoral course on social work intervention research, and the intervention has been informed by his training and practice experience related to LGBTQ youth, CBT, and group work. The BOWS intervention is based on several assumptions: (1) LGBTQ people are not abnormal but represent normal and natural variation in sexuality and gender among humanity; (2) social systems of oppression facing LGBTQ people (i.e., heterosexism and cisgendersim) are pervasive in society; (3) these systems of oppression lead to added stress for LGBTQ people in terms of hostile environments, negative interpersonal interactions, and discriminatory or inequitable policies and institutional systems; (4) because heterosexism and cisgenderism are pervasive in society, LGBTQ internalize heterosexist and cisgenderist attitudes and beliefs, which negatively affect their mental health; (5) this internalized oppression is a primary contributing factor to depression among LGBTQ people; and (6) interventions aimed at modifying cognition (e.g., CBT) can be used to target internalized oppression. 

Like CBT delivered on a one-on-one basis, group CBT is also effective at treating depression, according to meta-analytic findings with the general population [44,45]. In adapting general CBT for LGBTQ young people, the first author followed published practice-based recommendations by scholar-practitioners in this area [46,47,48,49,50]. BOWS was designed as a small group intervention (i.e., about 8 people per group). Group interventions are often most beneficial when there is a common problem focus (e.g., substance abuse, bereavement, and depression) and common identity (e.g., women, adolescents, and LGBTQ people). Unlike one-on-one psychotherapy or counseling, group interventions entail mutual social support, challenge feelings of isolation, and are cost-efficient. 

The primary goal of BOWS is to reduce depressive symptoms and promote mental health. Intervention sessions were designed to progressively build knowledge and skills among the participants. Table 1 shows an overview of the manualized BOWS sessions.

### 3.4. Data Collection

Data were collected from LGBTQ young adults using an online survey. To assess *demand* for the intervention, young adult participants completed the Short Mood and Feelings Questionnaire (SMFQ) [51], which is an 11-item depressive symptom scale where participants rated the extent they had experienced each item/symptom (e.g., “I didn’t enjoy anything at all”) in the past 2 weeks using a 3-point Likert-type scale ranging from 0 (*not true*) to 2 (*true*). The SMFQ has good evidence of reliability and validity [51,52,53]. The internal consistency reliability of this scale in this study was very good (α = 0.91). Scores from this measure allowed us to ascertain the proportion of youth experiencing depressive symptoms. To assess *acceptability*, young adults were given a 1-paragraph description of BOWS (see below) and then asked if they would be interested in participating in the intervention as part of an intervention trial study.

“Now we want to tell you about a potential program designed to reduce feelings of depression and promote mental health among lesbian, gay, bisexual, transgender, and queer (LGBTQ) young people. It’s called Being Out With Strengths (BOWS). A group of about 8 LGBTQ young adults (aged 18 to 29 years) will meet once a week for about 90 min for 8 weeks. The group will be led by a mental health professional. Young adults who participate will share their experiences, including some of the difficult aspects of being LGBTQ. Young adults will do activities to reflect on their thoughts and feelings and develop strategies for dealing with feelings of depression. By the end of the program, we hope that young adults will have made some friends in the group and have developed a sense of personal mental strength. The program would be free and part of a study.”

They were also asked if they had friends who could benefit from the intervention. To plan for potential future *implementation*, youth were asked where the intervention should take place (e.g., community center and outpatient office). Young adults were also asked times of day when they would be able to attend intervention sessions. 

Data were collected from mental health service providers using one-on-one semi-structured interviews that were audio-recorded. Questions related to *demand* included: Do you think that depression is a problem among LGBTQ young people? Do you think there is a need for an evidence-informed intervention like BOWS? Do you think the BOWS intervention could potentially have an impact on reducing depression among LGBTQ young people? Questions related to *acceptability* included: How likely do you think that LGBTQ young people experiencing some level of depression would participate in the BOWS intervention? Who would the intervention be appropriate for in terms of age, identity, and symptom severity? Do you think the number of BOWS sessions and length of each session is appropriate? Do you have any recommendations for improving the BOWS intervention? Questions related to *implementation* included: What settings do you think would be appropriate to deliver the BOWS intervention? Do you foresee any potential barriers to implementing the BOWS intervention? What is the ideal group size? What would be ideal in terms of the number of facilitators, their training, and their identity? Providers were prompted to elaborate on their responses when needed.

### 3.5. Data Analysis

Given the descriptive purpose of the study, quantitative survey data were analyzed using descriptive statistics, including sums, percentages, and means, as well as bivariate analyses. Depression scores were summed and a cut-off score of 8 was used to discern clinically significant depressive symptoms among young adults, which is based on prior studies showing sensitivity of 60–75% and specificity of 74–85% [51,54]. Qualitative data were transcribed and analyzed using qualitative content analysis with a conventional approach, which aims at description and allows themes to emerge from the data [55]. Following this approach, the first two authors independently read and wrote analytic memos about the text data. Second, the first two authors met and compared notes, discussed potential codes and categories, and derived a coding scheme. The coding scheme was organized using the three feasibility variables of demand, acceptability, and implementation as overarching categories. Third, the authors independently reread the qualitative responses and coded the text data using the established coding scheme. Fourth, the authors met to compare the results of their coding and resolved any discrepancies through negotiated consensus. 

Several strategies for rigor were used to help ensure that the qualitative findings were valid and trustworthy: triangulation, peer debriefing and support, and use of an audit trail. First, investigator triangulation was used because multiple authors coded and analyzed the data, which provides cross-checking of data by investigators. Second, interdisciplinary triangulation was used because the investigators represented the disciplines of psychology, social work, and public health, which led to a comprehensive understanding and interpretation of the data. Third, peer debriefing and support were used where the first 2 authors coding the data met with the third author to discuss challenges and issues related to the data and coding, to solicit feedback, and to offer new ideas or alternative perspectives. Finally, an audit trail was created, which included the raw data, initial analytic memos, notes from discussions about codes and categories, the final coding scheme, and notes from discussions about coding issues and questions.

## 4. Results

### 4.1. Demand

#### 4.1.1. Demand Results from Young Adults

Summed depressive symptom scores for young adults showed that 95% of participants experienced at least one depressive symptom some of the time in the past 2 weeks. Using the cutoff score of 8, nearly half (46%) of youth had clinically significant depressive symptoms.

#### 4.1.2. Demand Results from Providers

When providers were asked if depression is a problem among LGBTQ young people, all nine of them indicated that it was, and four mentioned that depressive symptoms are often accompanied with anxiety symptoms. Providers also noted that these mental health problems often stem from a society that generally stigmatizes LGBTQ people (*n* = 5). For example, one professional stated that “the environment is really toxic.” All of the professionals perceived a need for the BOWS intervention, with one queer-identified provider stating “We need targeted interventions for queer people, run by queer people, made by queer people because we’re disproportionately facing suicide…we need targeted, culturally humble interventions…and they’re not happening.” In addition to the need for LGBTQ-specific intervention resources, 3 providers also mentioned the need for a “peer group” space so that youth can feel “connected” and be in a “safe space.” Finally, all providers indicated that BOWS could be impactful because of the mutual support that groups can provide and being able to work through thoughts, feelings, and experiences in a structured way. One professional stated that clients’ “primary path to wellness in mental health is being empowered with their own thinking.”

### 4.2. Acceptability

#### 4.2.1. Acceptability Results from Young Adults

When asked if they would be interested in participating in BOWS as part of a study, 66% of young adults affirmed their interest, 28% indicated “maybe”, and only 5% indicated no interest. Among those who affirmed an interest, 71% of them had clinically significant depressive symptoms. For those who indicated that they would not be interested, reasons given by young adults were not feeling a strong connection to the LGBTQ community (*n* = 3), their mental health challenges were not related to their LGBTQ identity (*n* = 2), the time commitment (*n* = 2), already being connected with a mental health provider or resources (*n* = 2), the age range for the group was too wide (*n* = 1), and hesitation to be in a psychotherapy group (*n* = 1). Bivariate analyses showed no statistically significant differences in interest in participating in BOWS by race/ethnicity, χ^2^(1, *N* = 72) = 0.12, *p* = 0.73); sex, χ^2^(1, *N* = 71) = 3.85, *p* = 0.09); sexual orientation, χ^2^(3, *N* = 74) = 5.81, *p* = 0.12); or gender identity, χ^2^(1, *N* = 71) = 2.44, *p* = 0.17). When asked if they had friends who might be interested in BOWS, 74% responded “yes” and 26% responded “no.”

#### 4.2.2. Acceptability Results from Providers

In terms of group members, providers felt that BOWS would be acceptable for those in late adolescence, emerging adulthood, and young adulthood (i.e., aged 15–30 years). Many providers felt that the intervention would be appropriate for youth struggling with mild to moderate symptoms, and those with severe mental health problems would likely need more intensive interventions (*n* = 5). Two providers also mentioned that some youth may not be open or comfortable with their identities and do not want to be labeled, and therefore, would not be at a place in their identity development that would correspond with BOWS. Providers were also asked about including transgender and genderqueer youth in groups with cisgender queer youth, and providers were split in their responses with four recommending to include transgender/genderqueer youth, and five stating that it would be ideal to have separate groups for transgender/genderqueer but that having mixed groups of LGBTQ young people could also be beneficial. Comments from one provider illustrate the tension between differentiation and commonality regarding gender identity and sexual orientation experiences: “Gender and sexuality are different things, even though some of the cultural messages are the same and internalizing some of those negative messages are the same.” Another provider noted that having a queer-focused group intervention that is not inclusive of transgender people could be perceived as “exclusionary.”

Professionals believed that LGBTQ young people would participate in BOWS once they are in a group (*n* = 9), but several providers also expressed that there may be challenges with recruiting young people into the intervention, which would require intentional advertisement and outreach (*n* = 3). With regards to the appropriate number of sessions and length, four providers recommended at least eight sessions and all (*n* = 9) recommended 90 min per session. Two providers mentioned that one or two sessions could be added to focus on additional skill development or to follow-up about how clients/patients are integrating the CBT techniques into their everyday lives.

Other acceptability-related results centered on some of the strengths of BOWS that would be beneficial to young people. The content of the intervention was a strength noted by many of the providers (*n* = 5). Activities were described as “engaging,” handouts as “useful,” and the structure would “give youth a tool to talk about difficult things in a safe way.” Providers also endorsed the focus of mental reframing of negative internalized societal views about being LGBTQ (*n* = 4). As stated by one professional, “This helps raise awareness about where their stories come from. Developmentally younger people have a tendency to internalize things and think things are their fault, but this really helps show the different layers of systemic oppression that are teaching kids these messages.” Many providers also noted the *group* aspect of BOWS as a strength, for example, one provider felt that a group space would provide for “empathic witnessing” of youth’s struggles and experiences but also being a place for mutual support and affirmation. Four professionals specifically noted that the intervention manual was accessible and user-friendly (e.g., “As a practitioner, this is super easy to follow”). A final strength of BOWS was the organization and flow of topics (*n* = 2). One provider stated that there is “intuitive movement from concept level to concept level.” 

Despite the strengths of BOWS noted by providers, they also recommended some changes to the intervention so that it would be more sensitive and impactful for young people. The most common recommendation was to make sure there is enough time for young people to process their experiences and discuss the material in the group (*n* = 6). Three providers also recommended altering some of the CBT language to be more sensitive: “harmful thoughts” instead of “negative thoughts,” “reframing” instead of “reprogramming,” “unhelpful thinking” instead of “cognitive distortions.” Several providers noted that it might be beneficial to add an additional session or two to focus on how youth are incorporating the skills into their everyday lives and how they are addressing barriers (*n* = 3). Other suggestions involved improvements to the structure of intervention content, including making sure to review and discuss CBT homework at the beginning of all sessions (*n* = 2) and modifying the thought log format to include thoughts, feelings, behaviors, and situations, as well as having an example already filled in on the thought log (*n* = 2). Another recommendation involved integrating content on intersectionality, such as how youths’ LGBTQ identity intersects with their other identities (e.g., race/ethnicity and class), leading to diverse experiences and challenges (*n* = 2).

### 4.3. Implementation

#### 4.3.1. Implementation Results from Young Adults

In terms of possible settings where BOWS sessions should be held, the majority of young adults chose a college/university campus (*n* = 63), followed by an LGBTQ community center (*n* = 46), an office of a mental health professional (*n* = 12), and religious organization (*n* = 4), a restaurant (*n* = 2), some place accessible by public transportation (*n* = 2), at personal homes (*n =* 1), online/virtual platform (*n =* 1), a public library (*n =* 1). One respondent noted the importance of making the college-based venues welcoming to high schoolers as well. When asked if they would be more likely to participate in BOWS if food was provided at session meetings, 62% reported “yes”, 24% reported “maybe”, and 14% reported “no.” Additionally, when asked about what time of day they would most likely attend BOWS sessions, the majority reported during the evening time (i.e., 18:00, 19:00, or 20:00) (*n* = 66), followed by late afternoon (i.e., 16:00 or 17:00) (*n* = 27), mid-afternoon (i.e., 14:00 or 15:00) (*n* = 8), and early afternoon (i.e., 12:00 or 13:00) (*n* = 4). 

#### 4.3.2. Implementation Results from Providers

Professionals mentioned clinical and non-clinical settings where BOWS could be implemented. High schools were the most frequently mentioned setting (*n* = 8), followed by university/college counseling centers (*n* = 6), LGBTQ community centers (*n* = 5), and outpatient clinics/offices or wellness centers (*n* = 4). The least mentioned settings were churches (*n* = 2) and hospitals (*n* = 1). When asked about the ideal size of BOWS groups, providers stated that 6–8 young people would be ideal, with a minimum of four individuals and a maximum of 10 group members. 

In terms of the facilitators of the intervention, providers stated that two facilitators would be ideal but one would be sufficient. Providers also recommended that facilitators be licensed mental health professionals or in training to become a mental health professional, with preparation in CBT and group work. Providers also stated that ideally, facilitators would be members of the LGBTQ community, but experienced allies could also be effective facilitators. The importance of facilitators being open about their identity and personal background with group participants was also mentioned (*n* = 2). 

A number of potential barriers to implementing BOWS were noted by providers. The most common barrier mentioned related to parents, in terms of parents not being supportive of their child participating in an LGBTQ group or youth not being out to their parents about their LGBTQ identity, which may complicate their ability to participate in BOWS (*n* = 4). Other potential barriers included stigma related to mental illness or being LGBTQ (*n* = 3), concern about confidentiality (*n* = 2), and logistical challenges (e.g., finding a good time to meet and transportation to sessions) (*n* = 2).

## 5. Discussion

This study examined three feasibility constructs (i.e., demand, acceptability, and implementation) of the BOWS intervention for LGBTQ young people. The findings are discussed according to the research questions the study sought to answer, which arose from the three feasibility constructs. Implications of the results for a potential future efficacy trial of BOWS are also discussed.

### 5.1. Demand

Results from the young adult survey and provider interviews demonstrate a demand for the BOWS intervention in the study catchment area. This is unsurprising given the national prevalence data showing over half of LGBTQ young people experienced depressive symptoms at significantly higher rates compared to cisgender heterosexual young people [3,4]. With about half of the young adults in this study meeting clinical thresholds for depression and nearly all young adults reporting at least one symptom of depression, this supports the need to develop and test interventions to effectively address LGBTQ mental health. A demand for BOWS can also be seen from the survey results showing that no respondents that had clinically significant depressive symptoms answered “no” to their interest in participating in BOWS as part of a study. While some young people may be hesitant to commit to a group intervention, those who could potentially benefit the most from the intervention all expressed some degree of interest in participation. 

In addition, BOWS may not only fulfill a need for mental health care, but the group intervention modality of BOWS may also fulfill a need in the LGBTQ youth community for opportunities for social connection. Given the two layers of stigma for youth who are LGBTQ and dealing with mental health problems, connecting with a group may foster relationship-building and a sense of community, while also reducing feelings of isolation and invisibility. Thus, there is a demand for the BOWS intervention from a mental health and social perspective. 

Finally, several providers mentioned that many of their young LGBTQ clients/patients experience co-occurring depression and anxiety. Indeed, there is evidence of comorbidity of major depression and general anxiety disorder in the general population and among sexual minorities, with studies showing that just over half of individuals diagnosed with depression also had an anxiety disorder [56,57,58]. Given this, a future trial of BOWS could use depressive symptoms as the primary outcome variable and general anxiety symptoms as a secondary outcome. This is potentially significant because those with comorbid disorders likely have more severe impairment and an intervention that remediates two classes of mental health disorders would be efficient. 

### 5.2. Acceptability

The results demonstrate that the BOWS intervention is likely acceptable for the target population, specifically those in the late adolescent to young adult age range who identify as LGBTQ, with mild or moderate depressive symptoms. This is fitting, given that the prevalence rate of a major depressive episode is highest among individuals in late adolescence and early adulthood [59]. Interventions delivered at the early onset of depressive symptoms could prevent the persistence or escalation of depression into adulthood. High rates of depression have been found among LGBTQ adults and older adults [60,61,62,63,64,65]. Given the identity focus of BOWS, it is probably not appropriate for youth who are experiencing confusion or are questioning their sexual identity. Prior research suggests that these youth are often not interested in LGBTQ-specific groups until they are further in the identity development process [66] and may need a separate intervention catered to those who are questioning, exploring, and initially self-labeling their sexual identity [67]. 

There are many identities within the LGBTQ community, including transgender and cisgender people. Although the LGBTQ community faces a common system of social oppression related to gender and sexuality, transgender people face specific stressors related to their socially stigmatized gender identity that cisgender queer people do not. Thus, some providers in the study suggested that it may be beneficial for transgender youth to have their own BOWS group, which may help in group forming and addressing transgender-specific mental distress. However, providers also noted that having mixed groups of LGBTQ youth could also be beneficial for all young people. Heterogeneity in the LGBTQ community could be harnessed as a strength where young people see commonalities and differences in experiences, develop greater understanding of and respect for the diversity in their community, and share a variety of interpretations of distress and how to move toward healing and well-being. In the AFFIRM pilot study, a CBT-based intervention was implemented and evaluated with a heterogeneous group of LGBTQ adolescents, and the findings showed high acceptability and reductions in depression for the overall sample and the transgender subsample [36,37]. 

Challenges in recruiting LGBTQ people in particular into psychotherapy groups have been noted in the literature [67]. Historically, homosexuality and transgender experiences have been pathologized in the mental health professions, accompanied with the use of harmful techniques (e.g., castration, electroconvulsive shock therapy, lobotomies, and psychiatric institutionalization) to “treat” or “cure” LGBTQ people [68,69]. Some professionals continue to engage in conversion or reparative therapy, despite evidence of harm [70,71,72] and resolutions by mental health professional associations (e.g., American Counseling Association, American Psychiatric Association, American Psychological Association, National Association of Social Workers) opposing such practices [73,74,75,76]. Given this, LGBTQ people may be apprehensive and mistrustful of mental health interventions targeting their community. Recruitment materials need to underscore the affirmative focus of the intervention, and referrals for BOWS can be solicited from providers and community organizations who are trusted by the LGBTQ community.

The findings also demonstrate that eight sessions, 90 min each, is likely acceptable from the perspectives of young people and providers. Eight to 12 sessions is typical for CBT group interventions, and 90 min per session is also common [44,45]. Research shows that 60–90 min sessions are optimal because short sessions may be insufficient and long sessions may fatigue participants [44]. 

Noted strengths of BOWS included the focus on transforming internalized oppression via cognitive restructuring and BOWS being a group intervention. A significant body of evidence has linked LGBTQ-specific internalized oppression and the outcome of depression [25,27,41,42], making it an appropriate target for intervention. In addition, group interventions offer advantages over individual psychotherapy because clients/patients can gain insight, skills, and support through listening, talking, and interacting with others facing similar life challenges. Further, group interventions are often beneficial for socially stigmatized clients/patients because they may experience isolation and can learn how others have coped with similar challenges. In addition, participation in a group does not preclude clients/patients from doing individual psychotherapy at the same time; such an arrangement can be complimentary and depends on the needs and desires of the client/patient.

### 5.3. Implementation

Many colleges and universities offer psychoeducational, support, and/or therapy groups to undergraduate and graduate students as part of their counseling and psychological services, thus, there are infrastructures in place where BOWS could be easily delivered. However, about 30% of high school graduates do not attend college [77]. LGBTQ community centers would not have this limitation because they provide services to the community broadly; however, LGBTQ centers are uncommon in rural areas and the Mountain-Prairie and South Central U.S. regions [38]. Delivering BOWS in high schools would be optimal in terms of reach because almost all adolescents attend school and there are school counselors and social workers who could deliver the intervention. BOWS would likely need to be delivered after school due to constraining schedules during the school day. 

Other implementation-related findings focused on the composition of group members and facilitators. Systematic reviews of CBT-based groups show an average group size of 8.5 clients/patients per group, with a range of 6 to 10 [44,45]. A group with less than five members is not recommended [78]. In terms of the number of facilitators, two has been shown to be beneficial for clients/patients; however, a group led by one facilitator is common due to resource constraints and efficiency purposes [79]. Very little research has examined how the sexual or gender identity of a facilitator of an LGBTQ therapy group may affect group dynamics, processes, and outcomes. Extant research shows that heterosexual facilitators can be effective with LGBTQ group interventions [66,80]. Regardless of facilitator identity, it is recommended that group leaders remain mindful of their privileged status or shared identity, consider potential biases regarding the subgroups of the LGBTQ community and take necessary steps to address any bias, and disclose their identity during initial screening interviews with potential clients/patients, which can build trust and openness [67,81].

Regarding potential barriers to BOWS implementation, parent-related issues were common. In recent decades, state laws have given minors autonomy to consent to treatment for sensitive and private matters, such as sexually transmitted infections, alcohol or drug use, and mental health problems, which would preclude the need for parental consent [82,83]. Nonetheless, young people may want to tell their parents about their participation in BOWS even though they do not technically need their consent because it is a mental health intervention. Challenges related to this could be problem-solved using a portion of time during the first session, or facilitators could assist youth individually as this issue comes up in initial screening interviews.

### 5.4. Study Strengths and Limitations

This study had several strengths. First, data were collected and presented from two groups of participants, LGBTQ young adults and mental health service providers, both of whom would be essential to the BOWS intervention. Second, the feasibility of BOWS was comprehensively assessed using the constructs of demand, acceptability, and implementation, which were drawn from the established and widely-used framework devised by Bowen and colleagues [43]. Third, the study focuses on an intervention for a prevalent and deleterious mental health disorder facing the LGBTQ community, and there is limited intervention research in this area. Fourth, the study was conducted in the South where there are many LGBTQ people, yet hostile social climates, limited resources, and limited research—most LGBTQ-related research has been conducted in the Northeast and West Coast, typically in large urban areas (e.g., New York City, Boston, Los Angeles, and San Francisco). 

Despite the strengths of this study, there are several limitations. First, the study used purposive sampling, and thus, the samples may not be representative of LGBTQ young people or mental health professionals across the Triangle area, North Carolina, or the Southeast. Second, the sample of mental health providers was small and may not reflect the broader provider population. Third, there may have been selection bias because youth who took the survey may have been particularly interested in LGBTQ mental health issues. Fourth, young adult perceptions of BOWS acceptability were based on reading a 1-paragraph description of BOWS, as opposed to a lengthier description of all eight BOWS sessions or actually participating in the sessions through a pilot study. Therefore, the findings should be interpreted cautiously. 

## 6. Conclusions

In conclusion, the results demonstrate a need for the BOWS intervention given the prevalence of depression facing LGBTQ young people and limited research to rigorously evaluate CBT interventions for this population. Findings also indicate that BOWS would likely be acceptable for LGBTQ-identified adolescents and young adults struggling with mild or moderate depression; nonetheless, acceptability data would need to be collected from youth in a future pilot study. Modifications to BOWS can be made to further improve the intervention; however, these modifications are minor and various strengths of the intervention were identified. Implementation-related findings can be used to inform the design of a future randomized controlled trial, thereby increasing the likelihood of successful implementation of BOWS. Next steps in this research could include a pilot study to assess other elements of feasibility for an intervention trial, such as recruitment, randomization, or outcome measurement, as well as an assessment of preliminary efficacy.

## Figures and Tables

**Table 1:** Descriptions of sessions of CBT (cognitive behavioral therapy)-based interventions targeting depression among LGBTQ (lesbian, gay, bisexual, transgender, or queer) young people.

ESTEEM (Effective Skills to Empower Effective Men) [31,32,33]	AFFIRM [36,37]	BOWS (Being Out with Strength)
Discussed primary mental, behavioral, and sexual health issues; building motivation to address those issues; and reviewing participants’ unique strengths as gay or bisexual men. Reviewed the impact of minority stress on health, specific manifestations of minority stress, and current coping strategies. Raised awareness of the emotional impact of early and ongoing forms of minority stress. Raised awareness of the behavioral impact of minority stress and taught mindful, present-focused reactions to minority stress. Raised awareness of the cognitive impact of minority stress and posed cognitive restructuring activities. Engaged participants in a review of the impact of emotions on mental, behavioral, and sexual health and personal emotion avoidance tendencies driven by minority stress. Focused on the impact of minority stress on behavioral avoidance with a focus on creating an emotional and behavioral avoidance hierarchy. Engaged participants in behavioral experiments in which previously avoided experiences were gradually confronted. Continued the graduated behavioral experiments with a focus on assertiveness training as a skill for coping with minority stress. Reviewed new cognitive, affective, and behavioral coping strategies and their application to future minority stress experiences.	Introduction to CBT and understanding minority stress. Understanding the impact of anti-LGBTQ attitudes and behaviors on stress. Understanding how thoughts affect feelings. Using thoughts to change feelings. Exploring how activities affect feelings. Planning to overcome counterproductive thoughts and negative feelings. Understanding the impact of minority stress and anti-LGBTQ attitudes and behaviors on social relationships. Developing safe, supportive, and identity affirming social networks.	Introduce the purpose and goals of BOWS, establish group guidelines, encourage group forming, and introduce basic CBT concepts (e.g., connections between thoughts, feelings, behaviors, and situations; automatic thoughts). Elicit LGBTQ coming out stories and facilitate story sharing, introduce the concepts of harmful and helpful thoughts, and discuss and identify patterns of negative thinking. Explore social messages received about being LGBTQ, reflect on and discuss the content of these messages, discuss the sources of these messages, and introduce the concept of LGBTQ-related internalized oppression. Introduce the concept of core self-beliefs, identify problematic core self-beliefs based on thought records, and begin to consider the origins of negative core self-beliefs, which are likely related to LGBTQ-related oppression. Identify the origins of negative core self-beliefs, connect negative self-beliefs with emotional states, and discuss how to make peace with harmful experiences related to social learning of negative self-beliefs. Discuss the accuracy of thoughts, introduce the strategy of thought stopping, introduce the strategy of creating balanced or helpful alternatives to harmful or unhelpful thoughts, and practice replacing harmful or unhelpful thoughts with helpful thoughts. Discuss the emotional needs of people, identify feeling loved or cared for as a key emotional need, and introduce the idea of self-love (i.e., loving and caring for oneself), which is especially needed for marginalized groups like LGBTQ people. Discuss self-affirming and self-loving thoughts, practice naming one’s positive qualities, create individualized self-affirmations, practice incorporating self-affirmations into one’s thinking, which can offset negative social messages about being LGBTQ.
